# Massachusetts

**Published:** 1896-12

**Authors:** 


*Massachusetts.* Secretary Sessions, of the State Board of Agriculture, replying to a question as to the value of Prof. Bang's method for the eradication of tuberculosis as adopted in Denmark, replied that the present state of public opinion here would not permit the sale of milk or dairy-products from animals even slightly affected, and hence it would not pay to keep such animals unless they were exceptionally valuable. The present system of testing and immediate slaughter is radical and quite expensive to the State, but it is doubtful whether if any half-way measures would be wise or effective in combating so great a danger.

